# Remediation Potential of *Ulva lactuca* for Europium: Removal Efficiency, Metal Partitioning and Stress Biomarkers

**DOI:** 10.3390/jox16010020

**Published:** 2026-01-24

**Authors:** Saereh Mohammadpour, Thainara Viana, Rosa Freitas, Eduarda Pereira, Bruno Henriques

**Affiliations:** 1 Department of Biology, University of Aveiro, 3810-193 Aveiro, Portugal; 2 LAQV-REQUIMTE-Associated Laboratory for Green Chemistry, Department of Chemistry, Campus de Santiago, University of Aveiro, 3810-193 Aveiro, Portugaleduper@ua.pt (E.P.);; 3 CESAM-Centre for Environmental and Marine Studies, University of Aveiro, 3810-193 Aveiro, Portugal

**Keywords:** macroalgae, rare earth elements, phycoremediation, metal partitioning, oxidative stress

## Abstract

As demand for rare earth elements (REEs) rises and environmental concerns about the extraction of primary resources grow, biological methods for removing these elements have gained significant attention as eco-friendly alternatives. This study assessed the ability of the green macroalga *Ulva lactuca* to remove europium (Eu) from aqueous solutions, evaluated the cellular partition of this element and investigated the toxicological effects of Eu exposure on its biochemical performance. *U. lactuca* was exposed to variable concentrations of Eu (ranging from 0.5 to 50 mg/L), and the amount of Eu in both the solution and algal biomass was analyzed after 72 h. The results showed that *U. lactuca* successfully removed 85 to 95% of Eu at low exposure concentrations (0.5–5.0 mg/L), with removal efficiencies of 75% and 47% at 10 and 50 mg/L, respectively. Europium accumulated in algal biomass in a concentration-dependent manner, reaching up to 22 mg/g dry weight (DW) at 50 mg/L. The distribution of Eu between extracellular and intracellular fractions of *U. lactuca* demonstrated that at higher concentrations (5.0–50 mg/L), 93–97% of Eu remained bound to the extracellular fraction, whereas intracellular uptake accounted for approximately 20% at the lowest concentration (0.5 mg/L). Biochemical analyses showed significant modulation of antioxidant defenses. Superoxide dismutase activity increased at 10 and 50 mg/L, while catalase and glutathione peroxidase activities were enhanced at lower concentrations (0.5–1.0 mg/L) and inhibited at higher exposures. Lipid peroxidation levels remained similar to controls at most concentrations, with no evidence of severe membrane damage except at the highest Eu level. Overall, the results demonstrate that *U. lactuca* is an efficient and resilient biological system for Eu removal, combining high sorption capacity with controlled biochemical responses. These findings highlight its potential application in environmentally sustainable remediation strategies for REE-contaminated waters, while also providing insights into Eu toxicity and cellular partitioning mechanisms in marine macroalgae.

## 1. Introduction

Europium (Eu), a light rare earth element (REEs) within the lanthanide series, possesses a relatively low atomic number (63) and larger ionic radius compared to heavy rare earth elements [1]. These structural features contribute to its reduced density (5.264 g/cm^3^) and soft Mohs hardness (~2), making it the least dense and most reactive of the lanthanides. Eu predominantly exists in the +3-oxidation state (Eu^3+^) in environmental and industrial contexts, while the divalent form (Eu^2+^), although less stable, exhibits strong red luminescence under UV light, making it useful in phosphor applications [2,3]. This unique luminescent behavior of Eu has led to its use in a wide range of fields [4]. The outstanding photophysical properties of Eu compounds have made them especially valuable in biomedical diagnostics, bioimaging [5], and immunoassays [6]. Additionally, Eu is used in the production of permanent magnets [7], high-resolution display technologies [8], radars, and LED lighting [9,10].

Due to its broad range of applications, Eu is increasingly introduced into aquatic environments through industrial and agricultural activities [11,12,13]. Although naturally present in trace levels in seawater, rainwater, and stream water, the intensifying industrial use of Eu has led to a significant rise in its environmental concentrations, especially near mining operation sites and industrial effluent discharges. Europium concentrations in seawater and surface water exhibit considerable variability, depending on natural factors and anthropogenic inputs [14,15,16,17]. For example, Eu levels in rivers in the Huainan coal mining area (Huainan City, Anhui Province, China) range from 27.9 to 35.8 ng/L [14], while in the Yongding River, concentrations vary from 0.27 to 1.74 ng/L [15]. In the Nida River watershed in south central Poland, values range from 6 to 11 ng/L [16], and in the tropical South Atlantic Ocean, Eu concentrations range from 0.68 to 2.02 pmol/kg [17]. In Switzerland, approximately 44 kg of Eu per year, or 0.005 g Eu per capita per year, enter various wastewater systems annually [18].

Once introduced into aquatic habitats, Eu can be taken up by aquatic organisms and accumulate to levels associated with measurable toxicity. Under laboratory conditions, Pinto et al. [19] assessed the acute toxicity of Eu using the standard aquatic species *Daphnia magna*, reporting EC_50_ values within the range of 5–100 mg/L. The primary effect observed was immobilization, indicating impaired swimming ability and subsequent mortality at higher concentrations. Leite et al. [20] showed that the mussel *Mytilus galloprovincialis* exposed to Eu (10–80 μg/L) accumulated the element in a dose-dependent manner and exhibited significant biochemical and histopathological alterations, including cellular damage at all Eu concentrations, while histopathological injuries were significant in digestive tubules at 10–40 μg/L and did not fully recover after 14 days in clean seawater. Extending this work, Leite et al. [21] exposed Eu at 10 μg/L under varying salinities (20, 30, 40) in adults and sperm of *M. galloprovincialis*. Adults were most affected when Eu acted in combination with high salinity (40), which altered defense behavior and led to redox imbalance and cellular damage; in sperm, Eu at high salinity significantly reduced the percentage of motile cells and increased irregular movement. These findings highlight that Eu exposure induces adverse effects across different levels of biological organization, from cellular stress and tissue damage in bivalves and whole-organism toxicity in crustaceans.

From an analytical perspective, the accurate determination of Eu in environmental and biological matrices relies on highly sensitive and selective techniques. Inductively coupled plasma mass spectrometry (ICP-MS) is the most widely used method due to its low detection limits, multi-element capability, and suitability for analyzing trace levels in complex aqueous systems, including seawater and biological tissues. Complementary approaches, such as inductively coupled plasma optical emission spectrometry (ICP-OES), are also employed for higher concentration ranges, while luminescence-based methods exploiting the characteristic photophysical properties of Eu^3+^ have recently gained attention for selective detection and speciation in environmental samples. Advances in sample preparation, separation strategies, and spectrometric detection continue to improve the reliability of Eu quantification in complex matrices, supporting both environmental monitoring and removal/recovery studies [22,23].

Given the critical demand for Eu, intensified by its diverse applications, mining alone is insufficient to meet global needs. Recycling and recovery strategies have therefore become essential [8,24,25]. Importantly, the scarcity of REEs is not attributed to their low natural abundance. Rather, this scarcity arises from the fact that REEs are rarely found in pure form in nature. Instead, they are typically dispersed and occur in combination with other elements, making their extraction complex and costly [8,26,27]. Various abiotic removal/recovery methods have been developed, each offering advantages and disadvantages: chemical bleaching is cost-effective but environmentally hazardous due to acidic effluents [28]; electrochemical methods are energy-efficient but contribute to waste generation [29]; and ionic liquids offer sustainability in liquid–liquid extraction but may pose toxicity risks [30]. In contrast, biological removal/recovery methods, especially bioremediation using algae, offer a safer and more environmentally friendly alternative [31]. Bioremediation offers a sustainable and economically viable strategy by using living organisms, including microorganisms, fungi, and green plants, or their enzymes to degrade, detoxify, or convert hazardous environmental pollutants into less harmful or inert forms [32]. Macroalgae, particularly *Gracilaria* and *Ulva* species, are excellent biosorbents for the removal of trace metals and REEs from wastewater due to their ability to absorb various components [32,33]. Their cell walls contain polysaccharides like alginates and ulvans, as well as proteins and sulfated sugars [32,34], which are rich in functional groups such as hydroxyl (–OH), carboxylic (–COOH), and amide (–NH). These functional groups facilitate ion exchange, electrostatic interactions, complexation, and microprecipitation, thus enabling efficient metal uptake [32,35]. Both living and non-living algae exhibit high biosorption capacity, offering a cost-effective and sustainable alternative to conventional treatment and removal/recovery methods [36]. Given this broad biosorption capacity, Henriques et al. [23] investigated Eu removal using *Ulva lactuca*, a green macroalga known for its strong sorption potential, once its sheet-like thallus provides a high surface-area-to-volume ratio, while its ulvan-rich cell walls contain abundant sulfate, carboxylate and hydroxyl functional groups that facilitate metal binding through ion-exchange and complexation, thereby enabling particularly efficient Eu uptake. Moreover, this macroalgae has demonstrated resilience in complex effluents containing multiple toxicants, further highlighting their suitability for real-world applications [37]. In this work, Henriques et al. [23] assessed the effects of Eu at concentrations of 10, 152 and 500 µg/L on several algal species, showing that *U. lactuca* achieved among the highest Eu removal rates (up to ~85% within 72 h). *U. lactuca* exhibited the greatest specific surface area (264 ± 31 cm^2^ g^−1^), which likely contributes to its superior efficiency in Eu removal compared with the other species studied.

Based on the existing evidence on REEs biosorption and toxicity in marine macroalgae, we hypothesized that *U. lactuca* can efficiently remove Eu from seawater across a broad concentration range, primarily through surface biosorption at higher exposure levels, while intracellular uptake becomes more relevant at lower concentrations. We further hypothesized that Eu exposure would elicit concentration-dependent biochemical responses, with antioxidant defense activation occurring prior to the onset of oxidative damage. Testing these hypotheses enables the simultaneous evaluation of *U. lactuca* as both a bioremediation tool and a biological model for assessing Eu toxicity and partitioning mechanisms. Despite increasing interest in the removal/recovery of REEs and ecotoxicology, important knowledge gaps remain regarding the behavior of Eu in marine primary producers. The existing studies have largely focused either on low, environmentally relevant concentrations or on removal efficiency alone, without simultaneously resolving extracellular versus intracellular partitioning and the associated biochemical responses. Moreover, information on the tolerance and detoxification mechanisms of marine macroalgae exposed to elevated Eu concentrations, representative of industrial or wastewater scenarios, remains scarce. By jointly assessing Eu removal efficiency, cellular partitioning, and oxidative stress biomarkers across a broad concentration range, the present study directly addresses this gap and provides an integrated framework linking bioremediation potential with ecotoxicological risk.

## 2. Materials and Methods

### 2.1. Reagents and Materials

All reagents used in this study were of analytical grade and sourced from reliable chemical suppliers. Europium(III) chloride hexahydrate salt (99.9%) was purchased from Thermo Scientific ™ (Waltham, MA, USA), and nitric acid (65% (*w*/*w*), Suprapur ^®^) was purchased from Merck (Darmstadt, Germany). All reagents used for biochemical analyses were of analytical grade or higher purity. Nitroblue tetrazolium (NBT), trichloroacetic acid (TCA), thiobarbituric acid (TBA), reduced nicotinamide adenine dinucleotide phosphate (NADPH), glutathione (reduced and oxidized forms), hydrogen peroxide (H_2_O_2_, 30%), bovine serum albumin (BSA), potassium phosphate buffers, sodium carbonate, sodium hydroxide, and formaldehyde standards were purchased from Sigma-Aldrich (St. Louis, MO, USA). Glutathione reductase and cumene hydroperoxide used in the glutathione peroxidase (GPx) assay were obtained from Sigma-Aldrich (St. Louis, MO, USA). Ethylenediaminetetraacetic acid (EDTA, ≥99%) and sodium chloride were supplied by Merck (Darmstadt, Germany). All solutions were prepared using ultrapure water (Milli-Q^®^, 18 MΩ/cm; Millipore, Burlington, MA, USA). Reagents were prepared fresh or stored according to the manufacturers’ recommendations to ensure analytical reliability. All standard solutions are metrologically traceable to SI units, as indicated on the manufacturer’s certificate of analysis. All laboratory materials and supplies were acid-washed following the protocol outlined by [23].

### 2.2. Collection and Preparation

The macroalgae *Ulva lactuca* were collected from the Ria de Aveiro (Portugal, 40°38′39″ N, 8°44′43″ W) during low tide and transported to the laboratory in isothermal bags containing natural seawater. Upon arrival, they were carefully rinsed with artificial seawater to decrease impurities such as animal feces and epiphytes. A small portion of the macroalgae was freeze-dried to determine the natural Eu concentration prior to the experiments. The required macroalgal biomass was cut into disks of Ø 5.5 cm for subsequent experiments and maintained in aerated aquariums filled with artificial seawater, kept at 20 ± 2 °C under a 12L:12D cycle.

Seawater was collected from the Atlantic Ocean near Praia da Barra in Aveiro, Portugal, and filtered through 0.45 µm Millipore filters. Subsequently, salinity was measured using an Eclipse model 45–63 portable refractometer (Bellingham + Stanley Ltd., Kent, UK) and diluted with ultrapure water (Milli-Q, 18 MΩ/cm) to a salinity of 10 g/L. This water was used to acclimate seaweed and to conduct assays.

### 2.3. Europium Accumulation by Ulva lactuca Macroalgae

The macroalgae were used at a concentration of 6 g/L (fresh weight (FW)) and placed in 1 L Schott flasks containing real seawater diluted to a salinity of 10 g/L, which was adjusted according to [37]. Europium was added to each flask as an aqueous stock solution (10,000 mg/L), prepared by dissolving europium(III) chloride hexahydrate in ultrapure water. The stock solution was subsequently used to contaminate the water in the experimental flasks to obtain the desired Eu concentrations (0.5, 1, 5, 10, and 50 mg/L). Although Eu concentrations in natural surface waters are typically reported in the ng to low µg/L range, substantially higher levels may occur locally in industrial effluents, mining-impacted waters, and waste streams associated with rare earth element processing [38,39]. In this context, the concentration range tested in the present study (0.5–50 mg/L) was selected to simulate contaminated and industrially relevant scenarios, enabling a robust assessment of removal efficiency, cellular partitioning, and the biochemical responses of *U. lactuca* under elevated Eu loads. Salinity and pH conditions were monitored and maintained at stable levels throughout the experimental period, so that their potential interactive effects could not be directly tested. Salinity (10 g/L) remained constant throughout the experimental period. In the controls (absence of macroalgae), pH remained relatively stable throughout the experiment, close to the initial value (7.8 ± 0.2). In treatments containing macroalgae, a slight increase in pH was observed, reaching approximately 8.5 ± 0.5 in the blanks (i.e., in the absence of Eu). In treatments with both Eu and macroalgae, pH generally increased to approximately 9.0 ± 0.5 within the first 6 h, remaining stable at lower Eu concentrations. However, at Eu concentrations ≥ 5 mg/L, slightly decreased over time, reaching values close to those of the controls by 72 h.

To distinguish abiotic losses of Eu from macroalgae-mediated removal, control flasks containing artificial seawater and Eu but no macroalgae were run in parallel. These controls provided the baseline against which algal uptake was evaluated. The system was left undisturbed for 24 h to ensure stabilization. Each treatment was conducted in triplicate, and water samples (approximately 10 mL) were collected at various times: 0, 1, 3, 6, 9, 24, 48, and 72 h [36]. Immediately after collection, water samples were acidified with HNO_3_ (Suprapur^®^, 65% (*v*/*v*)) to a pH ≤ 2 and stored at 4 °C. At the end of the 72 h, the macroalgae were freeze-dried to prepare them for subsequent analysis.

### 2.4. Extracellular and Intracellular Europium Concentrations in Ulva lactuca

The distribution of Eu between the extracellular surface (sorption) and intracellular regions (uptake) in *U. lactuca* was evaluated [36,40]. Algal samples exposed to Eu at concentrations of 0.5, 1.0, 5.0, 10, and 50 mg/L were washed with 0.001 mol/L Ethylenediaminetetraacetic acid (EDTA) in a 0.6 mol/L NaCl solution. A volume of 20 mL of each washing solution was used, and the samples and solutions were placed in Schott flasks and stirred for 15 min at 120 rpm. The pH of all washing solutions was adjusted to 8 to simulate seawater conditions. Finally, the concentration of Eu removed by the washing solution was considered an indicator of surface sorption, while the remaining concentration was attributed to intracellular uptake [36].

### 2.5. Measurement of Europium in Water and Macroalgae

The Eu concentration was measured using inductively coupled plasma optical emission spectrometry (ICP-OES) using a Jobin Yvon Activa M (HORIBA Jobin Yvon, Edison, NJ, USA) spectrometer. The coefficient of variation between triplicates was less than 5%. Only calibration curves with a correlation coefficient ≥ 0.999 were considered. For the determination of Eu concentrations in the water samples, the collected samples were directly analyzed. The limit of quantification was defined as the lowest calibration standard (100 µg/L), and the detection limit was considered 1/3 of the quantification limit (33 µg/L). An acid digestion step was required to measure the Eu concentration in macroalgae. Approximately 200 mg of the freeze-dried sample was weighed and placed into a Teflon reactor. Then, 2 mL of 65% HNO_3_, 0.25 mL of 30% H_2_O_2_, were added to the samples. The samples were then placed in a CEM Mars 6 microwave; the digestion process followed the protocol described by Henriques et al. [36]. A certified reference material (CRM), NIST 1515 (apple leaves, Eu 0.2 mg/kg), was used in parallel with the experiment, and the recovery rate was 95% [23,37].

### 2.6. Biomarker Analysis

After 72 h of exposure to Eu, the macroalgae were collected for biomarker analyses. The samples were prepared for biomarker determination by grinding them completely using a mortar and liquid nitrogen to achieve a regular consistency. The samples were then divided into four equal portions (0.25 g FW). For each biomarker analysis, a specific buffer was used: for lipid peroxidation (LPO), 20% trichloroacetic acid (TCA) was used, with a sample-to-buffer ratio of 2:1 (W:V); for enzyme activity determination, a potassium buffer was used. Following homogenization, the samples were transferred to microtubes containing the corresponding buffer. Microtubes were then placed in a QIAGEN^®^ TissueLizer II for 1 min to ensure complete tissue disruption. The homogenates were centrifuged at 4 °C for 25 min at 10,000× *g* to remove cellular debris. After centrifugation, the pellets were discarded, and the resulting supernatants were collected for subsequent biomarker analyses. Supernatants were either immediately used or stored at −80 °C until further analysis, following the procedure described by [23,41].

#### 2.6.1. Antioxidant and Biotransformation Activity

The activity of superoxide dismutase (SOD) was determined according to the method described in [42]. A calibration curve was prepared using SOD standards (0.25–60 U/mL). Absorbance was read at 560 nm. SOD activity was expressed in U per g FW, where U corresponds to a 50% inhibition of nitroblue tetrazolium (NBT) reduction.

CAT activity followed the procedure of Johansson and Borg [43], with modifications as proposed by Carregosa et al. [44]. Formaldehyde standards ranging from (0–150 μM) were used to produce the calibration curve, and absorbance readings were taken at 540 nm. Activity was expressed in U per g FW, where U reflects the enzymatic production of 1.0 nmol formaldehyde per min.

The activity of glutathione peroxidase (GPx) was quantified following the protocol of Paglia and Valentine [45]. Absorbance was recorded at 340 nm at 10 s intervals during 5 min, and enzymatic activity was calculated using the molar extinction coefficient (ε) of 6.22 mM^−1^ cm^−1^. GPx activity was expressed as U per g FW, where U corresponds to the amount of enzyme that catalyzes the oxidation of 1.0 μmol of NADPH per min.

#### 2.6.2. Cellular Damage

Levels of lipid peroxidation (LPO) were measured according to Ohkawa et al. [46], with absorbance read at 532 nm using the ε of 156 mM^−1^ cm^−1^. The results were reported as malondialdehyde (MDA) equivalents in nmol per g of FW.

### 2.7. Data Analysis

The amount of Eu removed from the solution by the seaweed (%) was calculated based on Equation (1):

(1) $$R(\%)=\frac{{(C}_{0}-C_{t})}{C_{0}}\times 100$$

where *C*_0_ is the initial concentration of Eu in solution (mg/L), and *C_t_* is the concentration of Eu at time *t* (mg/L). To facilitate comparison across assays involving different initial concentrations, the data were expressed as normalized concentrations, calculated as the ratio between the *C_t_* and the *C*_0_, i.e., *C_t_*/*C*_0_.

The average amount of element sorbed by the macroalga per unit mass, *q_t_* (mg/g, dry weight (DW)), after 72 h, was calculated via mass balance (Equation (2)):

(2) $$q_{t}(mg/g,\;DW)=\frac{(C_{0}-C_{t})\times V}{M}$$

where *M* (g) is the mass of macroalgae in *DW* and *V* (L) is the volume of solution.

The extracellular fraction of the element (*F_ext_*, %) was measured according to Equation (3):

(3) $$F_{ext}(\%)=\frac{q_{f}-q_{f\;EDTA}}{q_{f}}\;\times \;100$$

where *q_f_* is the total Eu concentration in *U. lactuca*, and *q_f_ EDTA* corresponds to the remaining Eu concentration after washing with EDTA. Then, to determine the intracellular concentration of Eu (*F_int_*, %) was calculated using Equation (4):

(4) $$F_{int}\left(\%\right)=100-F_{ext}$$

Biomarker data were analyzed using PRIMER v6 with PERMANOVA+ to test for differences among treatments [47]. Statistical significance was assessed using *p*-values, with *p* < 0.05 as the threshold.

## 3. Results

### 3.1. Europium Concentrations in Water

The concentration of Eu in almost all experimental conditions was close to the nominal values (0.5 to 50 mg/L), with a relative error of less than 20%. The largest relative deviation was observed at the 50 mg/L condition. In the blank, Eu concentration in water was below the detection limit (<0.020 mg/L, Table 1).

### 3.2. Efficiency of Ulva lactuca in Reducing Europium Levels in Seawater

Without *U. lactuca* (control), the concentration of Eu (0.5, 1.0, 5.0, 10 and 50 mg/L) in the solution remained stable over the 72 h, varying only between 3 and 16%; so no significant removal occurred in the solution under these conditions; the lack of a decrease in Eu in the control indicates that Eu was neither sorbed in its surroundings nor precipitated. In contrast, when macroalgae were present, Eu concentrations in seawater decreased significantly. Notably, at the lower concentrations of 0.5, 1.0, and 5.0 mg/L, considerable Eu removal was observed, ranging from 85 to 95% after 72 h. At higher concentrations of 10 and 50 mg/L, the observed removals were still significant, though slightly lower, at 75% and 47%, respectively.

**Figure 1:**
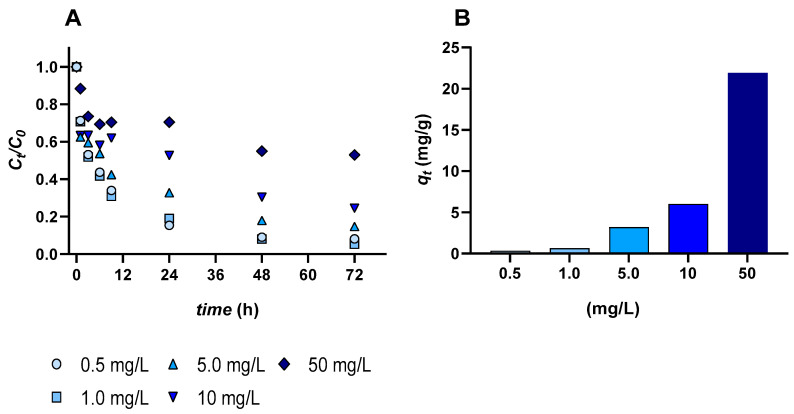
(**A**) Normalized concentration of europium (Eu) in solution over the 72 h exposure to *Ulva lactuca*; (**B**) concentration of Eu in the macroalgal biomass after exposure to Eu.

A reduction in Eu concentration was observed over time. The decrease occurred mainly during the initial hours, followed by a slower phase until equilibrium was reached (Figure 1A). Based on the kinetic profile, after approximately 9 h, the C_t_/C_0_ value at a concentration of 5 mg/L decreased to approximately 0.4, corresponding to a removal of 58%.

Quantifying the baseline Eu levels in the freshly collected macroalgal biomass showed that concentrations were below the quantification limit of 1.3 μg/g. At the end of the 72 h exposure period, Eu concentrations in the macroalgae showed a direct relationship with the water-exposure concentration, with the highest Eu enrichment (22 mg/g) occurring at 50 mg/L (Figure 1B). In the experiment with Eu, no signs of deterioration in *U. lactuca* were observed at any concentration.

### 3.3. Europium Partitioning in Ulva lactuca

**Figure 2:**
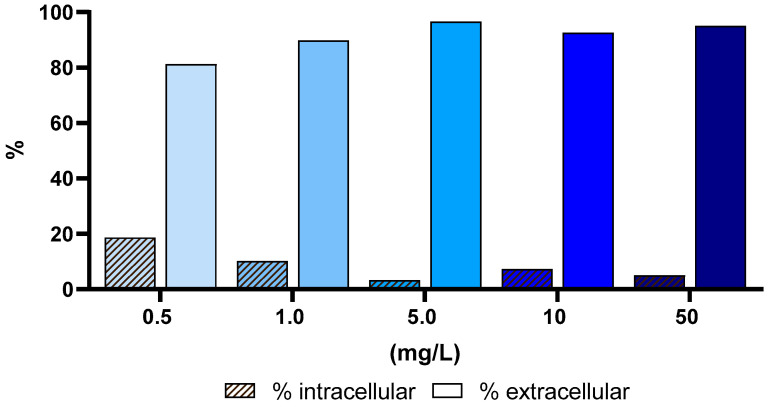
Intracellular and extracellular fractions of Eu in the macroalga *Ulva lactuca* after 72 h of exposure to different concentrations (0.5, 1.0, 5.0, 10 and 50 mg/L), operationally defined by extraction with EDTA 0.001 mol/L in NaCl 0.6 mol/L.

An EDTA solution was used to differentiate between surface sorption and internal uptake of Eu in *U. lactuca.* For exposure concentrations of 5.0, 10, and 50 mg/L, 97, 93 and 95% of the Eu removed from the solution were in the extracellular fraction of the macroalga, respectively (Figure 2). The lowest exposure concentration (0.5 mg/L) was the one at which the highest internal uptake of Eu by *U. lactuca* occurred, approximately 20%.

### 3.4. Biochemical Responses of Europium Toxicity in Ulva Lactuca

#### 3.4.1. Antioxidant Defenses

**Figure 3:**
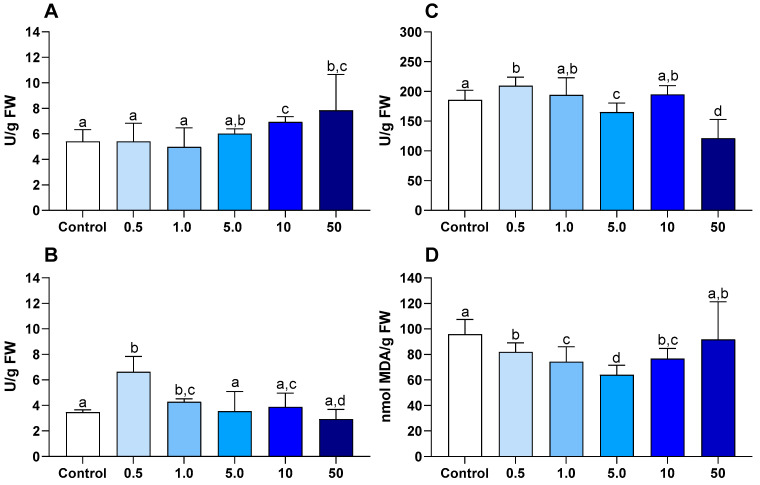
(**A**) Superoxide dismutase (SOD) activity; (**B**) catalase (CAT) activity; (**C**) glutathione peroxidase (GPx) activity; (**D**) lipid peroxidation (LPO) levels, in *Ulva lactuca* under control and different concentrations of Eu (0.5, 1.0, 5.0, 10, and 50 mg/L) for 3 days (72 h). The results are presented as mean ± standard deviation (n = 9). Different letters represent significant differences (*p* < 0.05) between the conditions.

The activity of SOD showed a significant increase at the concentrations of 10 and 50 mg/L, while no significant differences were observed at lower concentrations compared to the control (Figure 3A).

The activity of CAT increased significantly at the lowest concentrations of exposure (0.5 and 1.0 mg/L) compared to the control, while at higher concentrations, no significant differences were observed compared to the control (Figure 3B)

The activity of GPx increased significantly at the lowest concentration (0.5 mg/L) compared to the control, whereas at 5 and 50 mg/L, the activity decreased significantly (Figure 3C).

#### 3.4.2. Cellular Damage

In all treatments, LPO levels were significantly lower than in the control, except at the highest concentration (50 mg/L), where LPO levels did not differ significantly from the control (Figure 3D).

## 4. Discussion

The global demand for rare earth elements (REEs) continues to rise, driven largely by their essential applications in renewable energy systems, electronics, and advanced manufacturing. At the same time, concerns over the environmental impacts associated with mining and refining primary REE resources have intensified, making the recovery and reuse of these elements increasingly critical [48]. The excessive release of REEs into aquatic environments, whether from industrial effluents, waste mismanagement, or leaching from mine tailings, can contaminate water, negatively impact aquatic plants and animals, and disrupt trophic interactions. This is particularly concerning given the current lack of comprehensive toxicity data for many REEs in marine and freshwater species [49]. Although progress has been made in understanding the geochemical behavior of REEs, their biological effects across different trophic levels remain poorly constrained, especially under realistic environmental exposure scenarios.

To mitigate both resource depletion and ecological impacts, the development of effective removal, recovery and remediation strategies has become a central research priority. Conventional physico-chemical removal/recovery methods, such as solvent extraction, ion exchange, chemical precipitation, and electrochemical separation, can achieve high efficiencies but are often energy-intensive and costly, and they generate secondary waste streams that require further treatment. In contrast, biologically based strategies, including biosorption, bioaccumulation, microbial precipitation, and the use of functionalized biomaterials (e.g., algal biomass, bacterial consortia, biochar composites), offer promising alternatives [50]. These biological approaches can operate under mild conditions, exhibit high selectivity toward specific REEs ions, and produce minimal hazardous by-products. Moreover, they hold potential for integration into circular economy frameworks, enabling the removal of REEs from industrial effluents, electronic waste leachates, and mining wastewater while simultaneously reducing environmental impact. As research advances, optimizing these bioprocesses, particularly with respect to scalability, stability in saline and variable pH environments, and recovery of adsorbed metals, will be key to transitioning from laboratory systems to industrial-scale REEs circularity [51].

To address this knowledge gap, the current study focused on the removal and uptake of Eu at different concentrations by the green macroalga *U. lactuca*. The primary objective is to assess the biochemical responses of this macroalga to Eu exposure, providing valuable insights into its potential for environmental remediation and Eu recovery from waters.

### 4.1. Efficacy of Europium Load on Seawater Decontamination

Increasing the initial concentration of a contaminant generally provides a stronger driving force, helping overcome mass transfer resistances between the solution and the sorbent and thus enhancing contaminant sorption [23]. This physicochemical process occurs naturally on the surface of the macroalgae and does not require any external energy input [52]. Macroalgae effectively remove pollutants from water through physical and chemical interactions, making it a sustainable and efficient method for water decontamination. This process relies on algae’s natural ability to sorb pollutants, making it an attractive, eco-friendly alternative for treating contaminated water. In the initial phase of the experiment, a clear difference was observed between the pollutant concentration in the solution and its concentration in *U. lactuca.* The macroalgae exhibited more efficient bioremediation at lower concentrations, while the removal rate decreased at higher Eu levels. Although higher initial concentrations enhance interactions with active sites, they also accelerate the occupation of binding sites, as the existing macroalgae’s binding sites become more saturated than new binding sites are created (growth rate). This was also observed in the study by Viana et al. [53] on yttrium (Y), in which concentrations of 0.5, 5.0, 50, and 500 mg/L were examined, and saturation occurred at 50 and 500 mg/L. In this study, after approximately 48 h, the Eu concentration in the water phase decreased from 5 to 0.7 mg/L (Figure 1A). However, as time progressed, the sorption rate gradually declined, reaching a near-equilibrium state after approximately 72 h. This trend indicates that most of the available binding sites on the algal surface became saturated as the dissolved Eu concentration decreased. The rapid initial uptake, followed by a slower phase, reflects the typical two-step biosorption pattern observed in macroalgae. A similar observation was made by Ishii et al. [54], who noted that Hg absorption by macroalgae slowed as the algae’s capacity to sorb the element became saturated. Furthermore, the Hg concentration gradient between the solution and the algae decreased, contributing to a reduction in the sorption rate over time.

In this study, *U. lactuca* demonstrated remarkable efficiency in removing Eu from contaminated waters. At lower Eu concentrations (0.5–5 mg/L), it removed 85–95% of the pollutant within 72 h. Furthermore, at the highest Eu concentration tested (50 mg/L), the macroalgae accumulated up to 22 mg/g of Eu. The high Eu removal efficiencies observed in the present study (85–95% at 0.5–5 mg/L) are comparable to, or exceed, those previously reported for living macroalgae exposed to rare earth elements. For instance, Henriques et al. [23] reported Eu removal efficiencies of up to ~85% by *U. lactuca* at environmentally realistic concentrations (10–500 µg/L), while Pinto et al. [55] documented removal rates between 60 and 90% for several REEs (Y, lanthanum (La), cerium (Ce), and Eu) after 72 h, using different seaweed species. The higher absolute Eu accumulation observed in the present work (up to 22 mg/g DW at 50 mg/L) reflects the use of elevated, industrially relevant concentrations, extending previous findings obtained mainly at µg/L levels. Previous studies have shown that not only differences in contaminant and sorbent concentrations affect the sorption rate, but the macroalgae’s physical structure also plays a role [23]. Therefore, *U. lactuca* may be considered one of the best options for pollutant sorption due to its unique characteristics, including a thin and expansive thallus that provides a large surface area for adsorption, the highest growth rate among other macroalgae species, tolerance to a wide range of salinities, ability to thrive in polluted environments, and adaptability to a wide pH range. These findings highlight the potential of *U. lactuca* as an eco-friendly, cost-effective solution for Eu removal, providing a sustainable alternative to expensive, environmentally harmful conventional industrial processes. This finding contrasts with non-biological methods, which are typically less effective at low concentrations. The natural concentration of REEs in aquatic environments rarely exceeds 1 mg/L [56]. Some studies have investigated non-biological methods, such as those reported by Ni’am et al. [57], that can extract high percentages of REEs; however, these methods are often costly and suitable only for higher concentrations. Furthermore, these methods can contribute to environmental pollution due to the use of chemical solutions [58]. Removing REEs from low concentrations in aquatic environments is challenging, expensive, and energy-demanding. In contrast, this study highlights that *U. lactuca* can efficiently remove pollutants even at low concentrations, offering a more sustainable and profitable alternative to non-biological methods. However, it is essential to note that macroalgae’s ability to remove metals can decrease when concentrations exceed permissible limits. Henriques et al. [36] observed that *U. lactuca* was unable to reduce cadmium (Cd) (89 µg/L) and manganese (Mn) (454 µg/L) to acceptable levels. Thus, it is crucial to determine the permissible limits for REEs and the concentrations at which they cause toxicity and biochemical responses.

### 4.2. Europium Extra- and Intracellular Partition

The rapid initial decrease in Eu indicates surface sorption onto the macroalgae, driven by the concentration gradient between the external environment and the macroalgae’s interior. This process occurs passively, without requiring energy, as it follows the natural diffusion gradient [33,59]. Once the macroalgae surface becomes saturated with pollutants, further uptake occurs through membrane channels and protein pumps, which actively transport pollutants into the cell, including the cytoplasm, vacuoles, and inner membrane layers. This process requires energy and is carried out through cellular metabolism [40]. Based on this, *U. lactuca* was treated with an EDTA (0.001 mol/L) solution, which was chosen as the extraction agent because it was observed to minimize the risk of cell wall damage [36], as evidenced by the insignificant difference in potassium concentration before and after washing. At the lowest Eu concentration (0.5 mg/L), 20% of the pollutant was taken up intracellularly, further highlighting macroalgae’s ability to remove pollutants via active uptake mechanisms.

The predominance of extracellular Eu binding (>90% at 5–50 mg/L) is consistent with previous observations for REEs and other trivalent metals in macroalgae, where biosorption to cell wall functional groups is the primary uptake pathway. Similar extracellular dominance has been reported for Eu, Y and Gd in *U. lactuca* and *Gracilaria gracilis*, attributed to the abundance of negatively charged sulfate and carboxyl groups in ulvan-rich cell walls [23,33,55]. In contrast, at the lowest Eu concentration (0.5 mg/L), intracellular uptake accounted for approximately 20% of total Eu, indicating that active transport mechanisms become relatively more important under low external metal loads. Comparable concentration-dependent shifts toward intracellular accumulation have been described for REEs in microalgae and bivalves, where lower exposure levels favor metabolic uptake and vacuolar sequestration rather than surface saturation [20,54].

### 4.3. Toxicological Effect of Europium in Ulva lactuca

This study showed that *U. lactuca* is resistant to Eu and did not experience any mortality or degradation under various contamination scenarios. This is a significant advantage for using this macroalga in bioremediation, as it demonstrates that it can be used in polluted environments without concerns about physiological damage or reduced pollutant-removal efficiency [23]. An ideal macroalga for removing Eu should sorb contaminants without harming its function and prevent their release back into the environment. The chronic effects of Eu on macroalgae at different concentrations remain unclear, but biochemical analyses can detect toxicity immediately [60].

As organisms are exposed to stress conditions, they produce Reactive Oxygen Species (ROS), which can damage cell membranes through LPO [61]. In this study, except at the highest Eu concentration (50 mg/L), LPO levels at 0.5–10 mg/L were not significantly different from the control, indicating no clear evidence of cell membrane injury across most treatments. Consistent with a targeted antioxidant response, only SOD activity increased significantly (at 10 and 50 mg/L). This pattern suggests that SOD plays a key role in the antioxidant defense system of *U. lactuca* under Eu-induced oxidative stress. The modulation of antioxidant enzymes observed in *U. lactuca* exposed to Eu is consistent with previous reports on metal-induced oxidative stress in macroalgae. In particular, the significant induction of SOD at higher Eu concentrations (10–50 mg/L) is consistent with studies showing SOD as a first-line defense against metal-induced superoxide radicals, including exposures to Cu, Cd, and REEs [36,62]. The activity of GPx did not significantly increase at the lowest concentration (0.5 mg/L) compared to the blank. At higher concentrations of Eu (5.0, 10, and 50 mg/L), CAT and GPx activities tended to decrease, except for CAT at the lower concentrations (0.5 and 1.0 mg/L), where an increase was observed. The stimulation of CAT and GPx at lower Eu concentrations, followed by inhibition or normalization at higher levels, suggests an adaptive antioxidant response that becomes constrained as exposure increases. Similar biphasic responses have been reported in *U. lactuca* and other macroalgae exposed to Cu, Zn and Gd, where low metal levels activate detoxification pathways, while higher concentrations impair enzymatic efficiency due to oxidative overload or metal–enzyme interactions [37].

Taken together, Eu exposure in macroalgae was reported to trigger defense activation without overt cellular damage, consistent with our observation that oxidative stress signaling can occur alongside limited LPO change and a dominant SOD response [23]. The absence of significant lipid peroxidation at most Eu concentrations contrasts with reports of pronounced membrane damage in aquatic invertebrates exposed to environmentally relevant Eu levels [20,21], highlighting macroalgae’s greater tolerance to Eu stress. Similar resilience has been described for *U. lactuca* exposed to other metals, where effective antioxidant activation prevented LPO despite substantial metal accumulation. While Eu has been shown to induce severe cellular and tissue-level damage in bivalves and crustaceans at µg/L concentrations [19,20], the present results indicate that *U. lactuca* can tolerate substantially higher Eu loads with limited biochemical disruption. This discrepancy likely reflects fundamental differences in uptake pathways, with macroalgae relying primarily on extracellular biosorption rather than systemic internal distribution, thereby reducing toxicological risk.

The present findings demonstrate that *U. lactuca* can activate a range of defense mechanisms to withstand the potential damage caused by Eu, although its tolerance depends strongly on exposure level and cellular localization. At low concentrations (0.5 mg/L), approximately 20% of Eu entered the cells, triggering antioxidant defenses such as GPx and CAT. This response is consistent with previous reports showing that once internalized, Eu can be detoxified through vacuolar sequestration, autophagy, and biomineralization into EuPO_4_ [63]. In contrast, at higher exposures (5–50 mg/L), over 90% of Eu remained bound to the extracellular matrix, reflecting the anionic, ulvan-rich cell wall of *Ulva*, whose sulfate and uronic acid groups promote surface biosorption and explain the rapid initial decline in dissolved Eu [64]. Intracellular and extracellular fractions were differentiated using EDTA (0.001 M); the stable potassium levels before and after washing confirmed minimal cell-wall damage, while the absence of Eu loss in blanks ruled out precipitation or vessel adsorption. These results collectively indicate that while *U. lactuca* exhibits notable resilience through both extracellular binding and intracellular detoxification, Eu can still pose a significant threat, particularly at higher concentrations [23,65]. Moreover, linking the cellular localization of Eu with the corresponding enzymatic response provides a mechanistic framework useful for both ecotoxicological risk assessment and water-treatment optimization, helping to predict when metabolism-driven intracellular defenses are likely to dominate versus conditions under which extracellular, compensatory oxidative mechanisms prevail [66].

## 5. Conclusions

This study investigated europium (Eu) bioaccumulation, intra- and extracellular partitioning, and biochemical responses in *U. lactuca* exposed to concentrations ranging from 0.5 to 50 mg/L. The results demonstrated strong potential for *U. lactuca* to remove Eu within the studied range (0.5–50 mg/L), achieving removal efficiencies of up to 95% within 72 h at lower exposure levels (0.5–5.0 mg/L). Antioxidant enzymes such as SOD, CAT, and GPx were key components of the algal defense system, mitigating oxidative stress that became evident at higher Eu concentrations through increased ROS and MDA levels. This study emphasizes the double role of *U. lactuca* as both a biosorbent and a model organism for studying REEs toxicity. Moreover, these findings suggest that macroalgae-based removal methods offer an environmentally friendly and cost-effective alternative to chemical extraction, though the inhibitory effects observed under high Eu exposure warrant further investigation into the underlying toxicological mechanisms, particularly during long-term and combined contaminant exposure.

When compared with previous REE remediation and ecotoxicological studies, the present work demonstrates that *U. lactuca* exhibits removal efficiencies comparable to or exceeding those reported in the literature, along with a remarkable capacity to regulate oxidative stress. By simultaneously resolving removal kinetics, cellular partitioning and biochemical responses across a wide concentration range, this study extends earlier findings that focused either on environmentally realistic concentrations or on removal efficiency alone, providing a more integrated understanding of Eu–macroalga interactions. Overall, the study underscores the need to define safe REE concentrations in aquatic systems and encourages future research on the molecular basis of macroalgal responses and the optimization of bioaccumulation processes for large-scale environmental applications.

## Figures and Tables

**Table 1:** Nominal and measured concentrations in solutions under the various exposure treatments.

**Nominal Concentration (mg/L)**					
Blank	0.5	1.0	5.0	10	50
**Measured concentration (mg/L)**					
<0.020	0.41 ± 0.04	0.74 ± 0.02	4.1 ± 0.04	8.7 ± 0.49	50 ± 4.9

## Data Availability

The original contributions presented in this study are included in the article. Further inquiries can be directed to the corresponding author.

## References

[1] Aide M.T., Aide C. (2012). Rare earth elements: Their importance in understanding soil genesis. Int. Sch. Res. Not..

[2] Jarocka A., Fetliński B., Dębowski P., Pietrzak T.K., Jurak K., Wasiucionek M. (2022). Facile and cost-effective technique to control europium oxidation states in glassy fluorophosphate matrices with tunable photoluminescence. Sci. Rep..

[3] Miyamoto Y., Uekawa M., Ikeda H., Kaifu K. (1999). Electroluminescent properties of a Eu-complex doped in phosphorescent materials. J. Lumin..

[4] Lewandowski E.C., Arban C.B., Deal M.P., Batchev A.L., Allen M.J. (2024). Europium (II/III) coordination chemistry toward applications. Chem. Commun..

[5] Syamchand S.S., Sony G. (2015). Europium enabled luminescent nanoparticles for biomedical applications. J. Lumin..

[6] Evangelista R.A., Pollak A., Allore B., Templeton A.F., Morton R.C., Diamandis E.P. (1988). A new europium chelate for protein labelling and time-resolved fluorometric applications. Clin. Biochem..

[7] Coey J.M.D. (2020). Perspective and Prospects for Rare Earth Permanent Magnets. Engineering.

[8] Gonzalez V., Vignati D.A.L., Leyval C., Giamberini L. (2014). Environmental fate and ecotoxicity of lanthanides: Are they a uniform group beyond chemistry. Environ. Int..

[9] Rim K.T., Koo K.H., Park J.S. (2013). Toxicological Evaluations of Rare Earths and Their Health Impacts to Workers. Saf. Health Work.

[10] Ramos S.J., Dinali G.S., Oliveira O., Martins G.C., Moreira C.G., Siqueira O.J., Guilherme L.R.G. (2016). Rare Earth Elements in the Soil Environment. Curr. Pollut. Rep..

[11] Lafrenière M.-C., Lapierre J.-F., Ponton D.E., Guillemette F., Amyot M. (2023). Rare earth elements (REEs) behavior in a large river across a geological and anthropogenic gradient. Geochim. Cosmochim. Acta.

[12] Möller P., Knappe A., Dulski P. (2014). Seasonal variations of rare earths and yttrium distribution in the lowland Havel River, Germany, by agricultural fertilization and effluents of sewage treatment plants. Appl. Geochem..

[13] Banaee M., Mossotto C., Maganza A., Azizi R., Prearo M., Pastorino P., Faggio C. (2025). Rare earth elements on aquatic organisms: Toxicity, detoxification, and ecological implications. Emerg. Contam..

[14] Qian Y., Zheng L., Jiang C., Chen X., Chen Y., Xu Y., Chen Y. (2022). Environmental geochemical characteristics of rare-earth elements in surface waters in the Huainan coal mining area, Anhui Province, China. Environ. Geochem. Health.

[15] Gao X., Han G., Liu J., Zhang S. (2023). Spatial distribution and sources of rare earth elements in urban river water: The indicators of anthropogenic inputs. Water.

[16] Migaszewski Z.M., Gałuszka A. (2016). The use of gadolinium and europium concentrations as contaminant tracers in the Nida River watershed in south-central Poland. Geol. Q..

[17] Zheng X.-Y., Plancherel Y., Saito M.A., Scott P.M., Henderson G.M. (2016). Rare earth elements (REEs) in the tropical South Atlantic and quantitative deconvolution of their non-conservative behavior. Mar. Chem..

[18] Kaegi R., Gogos A., Voegelin A., Hug S.J., Winkel L.H.E., Buser A.M., Berg M. (2021). Quantification of individual Rare Earth Elements from industrial sources in sewage sludge. Water Res. X.

[19] Pinto I., Henriques B., Viana T., Freitas R., Pereira E., Antunes S.C. (2025). From high-tech to high-risk? Unveiling the acute ecotoxicological effects of rare earth elements on *Daphnia magna*. Bull. Environ. Contam. Toxicol..

[20] Leite C., Russo T., Pinto J., Polese G., Soares A.M.V.M., Pretti C., Pereira E., Freitas R. (2024). From the cellular to tissue alterations induced by two rare earth elements in the mussel species *Mytilus galloprovincialis*: Comparison between exposure and recovery periods. Sci. Total Environ..

[21] Leite C., Russo T., Polese G., Soares A.M.V.M., Pretti C., Pereira E., Freitas R. (2024). Effects of the interaction of salinity and rare earth elements on the health of *Mytilus galloprovincialis*: The case of praseodymium and europium. J. Xenobiot..

[22] Senila M., Levei E.A., Senila L., Cadara O. (2024). Validation of microwave acid digestion, diffusive gradients in thin-film preconcentration and inductively coupled plasma optical emission spectrometry methodology for the determination of REEs in natural zeolites. Anal. Methods.

[23] Henriques B., Morais T., Cardoso C.E.D., Freitas R., Viana T., Ferreira N., Fabre E., Pinheiro-Torres J., Pereira E. (2021). Can the recycling of europium from contaminated waters be achieved through living macroalgae? Study on accumulation and toxicological impacts under realistic concentrations. Sci. Total Environ..

[24] Costis S., Mueller K.K., Coudert L., Mihaela Neculita C., Reynier N., Blais J.F. (2020). Recovery potential of rare earth elements from mining and industrial residues: A review and cases studies. J. Geochem. Explor..

[25] Swain N., Mishra S. (2019). A review on the recovery and separation of rare earths and transition metals from secondary resources. J. Clean. Prod..

[26] Gkika D.A., Chalaris M., Kyzas G.Z. (2024). Review of methods for obtaining rare earth elements from recycling and their impact on the environment and human health. Processes.

[27] Opare E.O., Struhs E., Mirkouei A. (2021). A comparative state-of-technology review and future directions for rare earth element separation. Renew. Sustain. Energy Rev..

[28] Borja D., Nguyen K.A., Silva R.A., Park J.H., Gupta V., Han Y., Lee Y., Kim H. (2016). Experiences and Future Challenges of Bioleaching Research in South Korea. Minerals.

[29] Xu X., Sturm S., Samardzija Z., Scancar J., Markovicc K., Rozmana K.Z. (2019). A facile method for the simultaneous recovery of rare-earth elements and transition metals from Nd–Fe–B magnets. Green Chem..

[30] Huang C., Wang Y., Huang B., Dong Y., Sun X. (2019). The recovery of rare earth elements from coal combustion products by ionic liquids. Miner. Eng..

[31] Azubuike C.C., Chikere C.B., Okpokwasili G.C. (2016). Bioremediation techniques–classification based on site of application: Principles, advantages, limitations and prospects. World J. Microbiol. Biotechnol..

[32] Davis T.A., Volesky B., Mucci A. (2003). A review of the biochemistry of heavy metal biosorption by brown algae. Water Res..

[33] Jacinto J., Henriques B., Duarte A.C., Vale C., Pereira E. (2018). Removal and recovery of Critical Rare Elements from contaminated waters by living Gracilaria gracilis. J. Hazard. Mater..

[34] Cao Y., Shao P., Chen Y., Zhou X., Yang L., Shi H., Yu K., Luo X., Luo X. (2021). A critical review of the recovery of rare earth elements from wastewater by algae for resources recycling technologies. Resour. Conserv. Recycl..

[35] Herrero R., Lodeiro P., Rey-Castro C., Vilariño T., Sastre de Vicente M.E. (2005). Removal of inorganic mercury from aqueous solutions by biomass of the marine macroalga *Cystoseira baccata*. Water Res..

[36] Henriques B., Teixeira A., Figueira P., Reis A.T., Almeida J., Vale C., Pereira E. (2019). Simultaneous removal of trace elements from contaminated waters by living *Ulva lactuca*. Sci. Total Environ..

[37] Ferreira N., Ferreira A., Viana T., Lopes C.B., Costa M., Pinto J., Soares J., Pinheiro-Torres J., Henriques B., Pereira E. (2020). Assessment of marine macroalgae potential for gadolinium removal from contaminated aquatic systems. Sci. Total Environ..

[38] Fonseka C., Ryu S., Choo Y., Kandasamy J., Foseid L., Ratnaweera H., Vigneswaran S. (2024). Selective recovery of europium from real acid mine drainage using modified Cr-MIL and SBA15 adsorbents. Environ. Sci. Pollut. Res..

[39] Castro L., Gómez-Álvarez H., González F., Muñoz J.A. (2023). Biorecovery of rare earth elements from fluorescent lamp powder using the fungus *Aspergillus niger* in batch and semicontinuous systems. Miner. Eng..

[40] Hassler C.S., Slaveykova V.I., Wilkinson K.J. (2004). Discriminating between intra- and extracellular metals using chemical extractions. Limnol. Oceanogr. Methods.

[41] Freitas R., Silvestro S., Coppola F., Meucci V., Battaglia F., Intorre L., Soares A.M.V.M., Pretti C., Faggio C. (2020). Combined effects of salinity changes and salicylic acid exposure in *Mytilus galloprovincialis*. Sci. Total Environ..

[42] Beauchamp C., Fridovich I. (1971). Superoxide dismutase: Improved assays and an assay applicable to acrylamide gels. Anal. Biochem..

[43] Johansson L.H., Borg L.A.H. (1988). A spectrophotometric method for determination of catalase activity in small tissue samples. Anal. Biochem..

[44] Carregosa V., Velez C., Soares A.M.V.M., Figueira E., Freitas R. (2014). Physiological and biochemical responses of three Veneridae clams exposed to salinity changes. Ecotoxicol. Environ. Saf..

[45] Paglia D.E., Valentine W.N. (1967). Studies on the quantitative and qualitative characterization of erythrocyte glutathione peroxidase. J. Lab. Clin. Med..

[46] Ohkawa H., Ohishi N., Yagi K. (1979). Assay for lipid peroxides in animal tissues by thiobarbituric acid reaction. Anal. Biochem..

[47] Anderson M. (2008). PERMANOVA+ for PRIMER: Guide to Software and Statistical Methods.

[48] Ramprasad C., Gwenzi W., Chaukura N.C., Azelee N.I.V., Rajapaksha A.U., Naushad M., Rangabhashiyam S. (2022). Strategies and options for the sustainable recovery of rare earth elements from electrical and electronic waste. Chem. Eng. J..

[49] Malhotra N., Hsu H.S., Liang S.T., Roldan M.J.M., Lee J.S., Ger T.R., Hsiao C.D. (2020). An Updated Review of Toxicity Effect of the Rare Earth Elements (REEs) on Aquatic Organisms. Animals.

[50] Brown R.M., Mirkouei A., Reed D., Thompson V. (2023). Current nature-based biological practices for rare earth elements extraction and recovery: Bioleaching and biosorption. Renew. Sustain. Energy Rev..

[51] Heilmann M., Breiter R., Becker A.M. (2021). Towards rare earth element recovery from wastewaters: Biosorption using phototrophic organisms. Appl. Microbiol. Biotechnol..

[52] Naja G., Volesky B. (2008). Optimization of a Biosorption Column Performance. Environ. Sci. Technol..

[53] Viana T., Colónia J., Tavares D.S., Andrade M., Ferreira N., Freitas R., Pereira E., Henriques B. (2025). Uptake and effects of yttrium on the seaweed *Ulva* sp.: A study on the potential risks of rare earth elements in aquatic environments. Water.

[54] Ishii N., Tagami K., Uchida S. (2006). Removal of rare earth elements by algal flagellate *Euglena gracilis*. J. Alloys Compd..

[55] Pinto J., Costa M., Henriques B., Soares J., Dias M., Viana T., Ferreira N., Vale C., Pinheiro-Torres J., Pereira E. (2021). Competition among rare earth elements on sorption onto six seaweeds. J. Rare Earths.

[56] Atinkpahoun C.N.H., Pons M., Louis P., Leclerc J., Soclo H.H. (2020). Rare earth elements (REE) in the urban wastewater of Cotonou (Benin, West Africa). Chemosphere.

[57] Ni’am A.C., Wang Y., Chen S., Chang G., You S. (2020). Simultaneous recovery of rare earth elements from waste permanent magnets (WPMs) leach liquor by solvent extraction and hollow fiber supported liquid membrane. Chem. Eng. Process..

[58] Mwewa B., Tadie M., Ndlovu S., Simate G.S., Matinde E. (2022). Recovery of rare earth elements from acid mine drainage: A review of the extraction methods. J. Environ. Chem. Eng..

[59] Blaby-Haas C.E., Merchant S.S. (2012). The ins and outs of algal metal transport. Biochim. Biophys. Acta.

[60] Ma J., Wang W., Liu X., Wang Z., Gao G., Wu H., Li X., Xu J. (2020). Zinc toxicity alters the photosynthetic response of red alga *Pyropia yezoensis* to ocean acidification. Environ. Sci. Pollut. Res..

[61] Freitas R., Coppola F., Marchia L.D., Codella V., Prettib C., Chiellinic F., Morellic A., Polesed G., Soaresa A.M.V.M., Figueira E. (2018). The influence of Arsenic on the toxicity of carbon nanoparticles in bivalves. J. Hazard. Mater..

[62] Chen W., Sun M. (2024). Acute copper stress showed toxic effects on the physiological metabolism of *Ulva lactuca*, a common green macroalgae. Sci. Rep..

[63] Alonso P., Blas J., Amaro F., de Francisco P., Martín-González A., Gutiérrez J.C. (2024). Cellular response of adapted and non-adapted *Tetrahymena thermophila* strains to europium Eu(III) compounds. Biology.

[64] Li C., Tang T., Du Y., Jiang L., Yao Z., Ning L., Zhu B. (2023). Ulvan and *Ulva* oligosaccharides: A systematic review of structure, preparation, biological activities and applications. Bioresour. Bioprocess..

[65] Wang Y., Li Y., Luo X., Ren Y., Gao E., Gao H. (2018). Effects of yttrium and phosphorus on growth and physiological characteristics of *Microcystis aeruginosa*. J. Rare Earths.

[66] Wu L., Yang F., Xue Y., Gu R., Liu H., Xia D., Liu Y. (2023). The biological functions of europium-containing biomaterials: A systematic review. Mater. Today Bio.

